# Bis(1,3-benzothia­zol-2-amine-κ*N* ^3^)silver(I) nitrate acetone solvate

**DOI:** 10.1107/S1600536809004176

**Published:** 2009-02-11

**Authors:** Christoph E. Strasser, Leigh-Anne de Jongh, Stephanie Cronje, Helgard G. Raubenheimer

**Affiliations:** a Department of Chemistry and Polymer Science, University of Stellenbosch, Private Bag X1, Matieland 7602, South Africa

## Abstract

In the title compound, [Ag(C_7_H_6_N_2_S)_2_]NO_3_·C_3_H_6_O, the Ag^I^ ion is coordinated to two benzothia­zol-2-amine ligands *via* the thia­zole N atoms in an approximately linear arrangement. The dihedral angle between the mean planes of the two 1,3-benzothia­zole groups is 5.9 (3)°. Both amine groups on the ligands are oriented in the same direction and are engaged in N—H⋯O hydrogen bonding with the nitrate counter-anion, forming one-dimensional columns along the *b*-axis direction. Voids created by inefficient crystal packing are occupied by acetone solvent mol­ecules which are disordered over two sites with occupancies of 0.563 (11) and 0.437 (11).

## Related literature

For general background, see: de Jongh *et al.* (2008 ▶); Tewari *et al.* (1991 ▶). For related structures, see: Ellsworth *et al.* (2006 ▶); Fackler *et al.* (1992 ▶); Fitchett & Steel (2000 ▶); Hiraoka *et al.* (2003 ▶); Manzoni de Oliveira *et al.* (2007 ▶); Murthy & Murthy (1976 ▶); Zou *et al.* (2004 ▶).
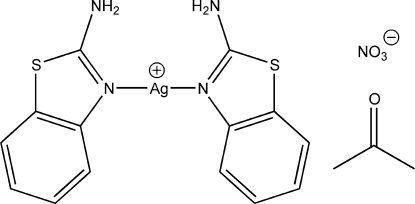


## Experimental

### 

#### Crystal data

[Ag(C_7_H_6_N_2_S)_2_]NO_3_·C_3_H_6_O
*M* *_r_* = 528.35
Monoclinic, 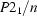

*a* = 17.096 (4) Å
*b* = 5.8166 (12) Å
*c* = 20.421 (4) Å
β = 102.867 (3)°
*V* = 1979.8 (7) Å^3^
*Z* = 4
Mo *K*α radiation
μ = 1.27 mm^−1^
*T* = 100 (2) K
0.20 × 0.08 × 0.05 mm

#### Data collection

Bruker APEX CCD area-detector diffractometer
Absorption correction: multi-scan (*SADABS*; Bruker, 2002 ▶) *T*_min_ = 0.786, *T*_max_ = 0.940
10935 measured reflections
4046 independent reflections
3594 reflections with *I* > 2σ(*I*)
*R*_int_ = 0.029

#### Refinement

*R*[*F* ^2^ > 2σ(*F* ^2^)] = 0.056
*wR*(*F* ^2^) = 0.133
*S* = 1.12
4046 reflections
259 parameters
12 restraints
H-atom parameters constrained
Δρ_max_ = 1.91 e Å^−3^
Δρ_min_ = −1.06 e Å^−3^

### 

Data collection: *SMART* (Bruker, 2002 ▶); cell refinement: *SAINT* (Bruker, 2003 ▶); data reduction: *SAINT*; program(s) used to solve structure: *SHELXS97* (Sheldrick, 2008 ▶); program(s) used to refine structure: *SHELXL97* (Sheldrick, 2008 ▶); molecular graphics: *X-SEED* (Atwood & Barbour, 2003 ▶; Barbour, 2001 ▶); software used to prepare material for publication: *X-SEED*.

## Supplementary Material

Crystal structure: contains datablocks I, global. DOI: 10.1107/S1600536809004176/lh2768sup1.cif

Structure factors: contains datablocks I. DOI: 10.1107/S1600536809004176/lh2768Isup2.hkl

Additional supplementary materials:  crystallographic information; 3D view; checkCIF report

## Figures and Tables

**Table:** 

Ag1—N13	2.130 (4)
Ag1—N23	2.127 (4)

**Table:** 

N13—Ag1—N23	171.84 (17)

**Table 2:** Hydrogen-bond geometry (Å, °)

*D*—H⋯*A*	*D*—H	H⋯*A*	*D*⋯*A*	*D*—H⋯*A*
N12—H2⋯O1	0.88	1.99	2.851 (6)	165
N12—H1⋯O2^ii^	0.88	2.07	2.889 (6)	155
N22—H3⋯O1	0.88	2.12	2.959 (7)	158
N22—H4⋯O1^iii^	0.88	2.11	2.955 (6)	162

Symmetry codes: (ii) 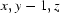; (iii) 
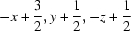
.

**Fig. 1.**
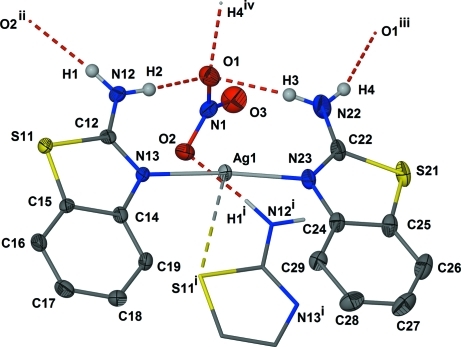
The asymmetric unit of (I), ellipsoids are drawn at the 50% probability level; the disordered acetone molecule and hydrogen atoms not involved in hydrogen bonding are omitted. A part of the symmetry-related 1,3-benzothiazole moiety that forms the Ag1···S11i contact is shown as a stick model. Symmetry codes: (i) = x, y + 1, z; (ii) = x, y–1, z; (iii) = –x+3/2, y + 1/2, –z+1/2; (iv) = –x+3/2, y–1/2, –z+1/2.

**Fig. 2.**
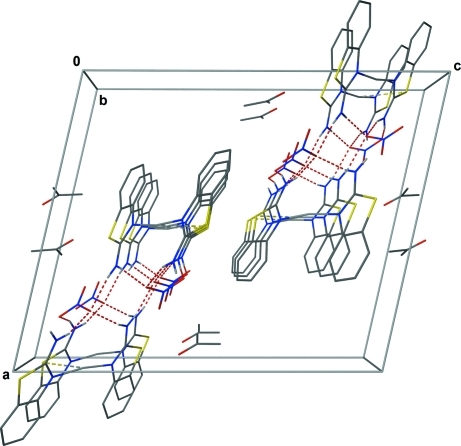
Perspective view of the crystal structure along the b axis. Only hydrogen atoms involved in hydrogen bonding (represented by dotted lines) are shown.

## supplementary crystallographic information

### Comment

The cation in the title compound (I) (Fig. 1) is crystallographically independent and consists of two benzothiazol-2-amine ligands coordinating to an Ag^I^ ion with their thiazole imine nitrogen atoms, thus furnishing an essentially linear geometry around the metal. This is in contrast to a postulation of amino-N coordination by Tewari *et al.* (1991) which was based on infrared evidence. However, hydrogen bonding may have interfered in the assignment of bands. Their conclusions that nitrate is not bonded to silver and the presence of Ag···S contacts have now been verified for the structure of (I).

The nitrate counter-anion in (I) does not interact with the metal which is reflected in the close to linear N—Ag—N angle of 171.84 (17)°. Similar molecular structures wherein the Ag^I^ ion is coordinated to two thiazole imine nitrogen atoms and interacts with nitrate, have markedly bent angles of 143.2 (2) and 146.1 (2)° (Fitchett & Steel, 2000), 136.05 (17) and 130.78 (15)° (Zou *et al.*, 2004). Perchlorate shows the same effect with an angle of 144.3 (6)° (Murthy & Murthy, 1976). Essentially linear angles of 173.5 (2), 176.1 (2) and 176.8 (2)° are, however, observed in a trinuclear silver complex (Hiraoka *et al.*, 2003) even though trifluoromethanesulfonate or methanol additionally coordinate to the silver atoms. The Ag—N bond lengths of 2.148 (6)–2.188 (6) Å in this complex are comparable to (I) [2.130 (4) and 2.127 (4) Å], as are the appropriate bond distances in the undisturbed (*i.e.* the Ag centre is only coordinated by thiazole ligands and the anion does not form part of the coordination sphere) silver perchlorate complexes of bis(benzothiazol-2-ylsulfanyl)methane and 1,4-bis(benzothiazol-2-ylsulfanyl)butane reported by Zou *et al.* (2004) [2.136 (4) and 2.147 (4) Å in the former and 2.136 (5) Å in the latter complex].

The planes of the two 1,3-benzothiazole groups in (I) lie at an angle of 5.9 (3)° which prevents crowding between H19 and H29 that would otherwise ensue in a flat cation. A short contact between Ag1 and S11^i^ [3.2261 (15) Å, symmetry code: (i) = *x*, *y* + 1, *z*] can be observed which is shorter than the sum of the van der Waals radii of the concerned atoms. Such Ag···S interactions involving thiazole rings have been observed before with distances of 3.306 Å (Ellsworth *et al.*, 2006), 3.336 Å (Fackler Jr *et al.*, 1992), 3.204 Å (Manzoni de Oliveira *et al.*, 2007) and 3.543 Å (Zou *et al.*, 2004); except for the longer distance in the last example they are comparable to (I).

The nitrate counteranion plays a crucial role in governing the crystal structure of (I). The amino groups of the cations engage in hydrogen bonds to the nitrate anion and form polar one-dimensional hydrogen bonding domains ordered around the crystallographical 2_1_ screw axes. The apolar 1,3-benzothiazole "ends" of different columns face each other as well as the co-crystallized acetone solvent molecules (Fig. 2). The hydrogen bonding network (Fig. 1) emanates from the two amino groups of the cation which chelate O1 of the nitrate anion as well as hydrogen bonding to two other nitrates, one from the same side of the chain (*via* O2, related by a translation in b) and one from the other side (*via* O1, related by a 2_1_ screw operation). O1 accepts three hydrogen bonds and O2 is involved in a single hydrogen bond. O3 does not exhibit hydrogen bonding which might be a cause of its larger thermal ellipsoid due to less restriction in movement.

The only possible π···π-interaction between the heteroaromatic rings is found in the 1,3-benzothiazole containing S11 and its counterpart generated by a centre of inversion [symmetry code: (v) = –x+2, –y+1, –z] with centroid–centroid distances of 3.89 Å.

The acetone solvent is highly disordered and occupies two sites in a 0.56:0.44 ratio. The carbonyl groups roughly point in opposite directions. Additional electron density peaks around the solvent as well as very high *U*_eq_ values suggest a high mobility of the acetone molecule.

### Experimental

The formation of crystalline (I) occurred during the reaction of [Au(NO_3_)(PPh_3_)] (0.20 g, 0.38 mmol) with benzothiazol-2-amine (57 mg, 0.38 mmol) in acetone solution (20 ml). It could be traced to the utilization of AgNO_3_ and [AuCl(PPh_3_)] in the preceding preparation of the gold reagent.

### Refinement

To obtain a satisfactory geometry, the bond lengths in both orientations of the acetone molecule were restrained to target distances (C=O 1.2 Å and C—C 1.5 Å) and the molecules themselves restrained to be flat. The occupancies for the A and B orientation refined to 0.563 (11) and 0.437 (11), respectively.

All H atoms were positioned geometrically (C—H = 0.95, 0.99 and 0.98 Å for CH, CH_2_ and CH_3_ groups, respectively; N—H = 0.92 Å) and constrained to ride on their parent atoms; *U*_iso_(H) values were set at 1.2 times *U*_eq_(C,*N*) except for methyl groups where *U*_iso_(H) was set at 1.5 times *U*_eq_(C).

The largest residual electron density peak of 1.91 e Å^-3^ is located 0.68 Å from O4A of the acetone solvent molecule, the largest hole of –1.06 e Å^-3^ is located 0.54 Å from O4B.

### Crystal data

**Table:**

[Ag(C_7_H_6_N_2_S)_2_]NO_3_·C_3_H_6_O	*F*(000) = 1064
*M**_r_* = 528.35	*D*_x_ = 1.773 Mg m^−^^3^
Monoclinic, *P*2_1_/*n*	Mo *K*α radiation, λ = 0.71073 Å
Hall symbol: -P 2yn	Cell parameters from 4102 reflections
*a* = 17.096 (4) Å	θ = 2.4–26.4°
*b* = 5.8166 (12) Å	µ = 1.26 mm^−^^1^
*c* = 20.421 (4) Å	*T* = 100 K
β = 102.867 (3)°	Needle, colourless
*V* = 1979.8 (7) Å^3^	0.20 × 0.08 × 0.05 mm
*Z* = 4	

### Data collection

**Table:**

Bruker APEX CCD area-detector diffractometer	4046 independent reflections
Radiation source: fine-focus sealed tube	3594 reflections with *I* > 2σ(*I*)
graphite	*R*_int_ = 0.029
ω scans	θ_max_ = 26.4°, θ_min_ = 1.8°
Absorption correction: multi-scan (*SADABS*; Bruker, 2002)	*h* = −16→21
*T*_min_ = 0.786, *T*_max_ = 0.940	*k* = −7→7
10935 measured reflections	*l* = −22→25

### Refinement

**Table:**

Refinement on *F*^2^	Primary atom site location: structure-invariant direct methods
Least-squares matrix: full	Secondary atom site location: difference Fourier map
*R*[*F*^2^ > 2σ(*F*^2^)] = 0.056	Hydrogen site location: inferred from neighbouring sites
*wR*(*F*^2^) = 0.133	H-atom parameters constrained
*S* = 1.12	*w* = 1/[σ^2^(*F*_o_^2^) + (0.0594*P*)^2^ + 10.016*P*] where *P* = (*F*_o_^2^ + 2*F*_c_^2^)/3
4046 reflections	(Δ/σ)_max_ = 0.001
259 parameters	Δρ_max_ = 1.91 e Å^−^^3^
12 restraints	Δρ_min_ = −1.06 e Å^−^^3^

### Special details

**Table:**

Geometry. All e.s.d.'s (except the e.s.d. in the dihedral angle between two l.s. planes) are estimated using the full covariance matrix. The cell e.s.d.'s are taken into account individually in the estimation of e.s.d.'s in distances, angles and torsion angles; correlations between e.s.d.'s in cell parameters are only used when they are defined by crystal symmetry. An approximate (isotropic) treatment of cell e.s.d.'s is used for estimating e.s.d.'s involving l.s. planes.
Refinement. Refinement of *F*^2^ against ALL reflections. The weighted *R*-factor *wR* and goodness of fit *S* are based on *F*^2^, conventional *R*-factors *R* are based on *F*, with *F* set to zero for negative *F*^2^. The threshold expression of *F*^2^ > 2σ(*F*^2^) is used only for calculating *R*-factors(gt) *etc*. and is not relevant to the choice of reflections for refinement. *R*-factors based on *F*^2^ are statistically about twice as large as those based on *F*, and *R*- factors based on ALL data will be even larger.The acetone molecule is disordered around two positions with the C—O vectors pointing in roughly opposite directions. The bonds were restrained to target distances (1.2 Å for C═O and 1.5 Å for C—C) and the molecules were restrained to be flat. Due to the heavy disorder, anisotropic refinement of the molecule was not possible.

### Fractional atomic coordinates and isotropic or equivalent isotropic displacement parameters (Å^2^)

**Table:**

	*x*	*y*	*z*	*U*_iso_*/*U*_eq_	Occ. (<1)
Ag1	0.98098 (2)	0.87563 (7)	0.191835 (19)	0.02109 (14)	
S11	0.98002 (9)	0.2069 (2)	0.06495 (7)	0.0230 (3)	
S21	0.94328 (10)	1.5059 (3)	0.32405 (7)	0.0335 (4)	
O1	0.7930 (2)	0.7687 (7)	0.1790 (2)	0.0320 (9)	
O2	0.8171 (3)	0.9490 (8)	0.0933 (2)	0.0407 (11)	
O3	0.7210 (3)	1.0619 (9)	0.1388 (3)	0.0458 (12)	
N1	0.7758 (3)	0.9271 (8)	0.1357 (2)	0.0270 (10)	
N12	0.8732 (3)	0.4138 (8)	0.1233 (2)	0.0254 (10)	
H2	0.8564	0.5233	0.1465	0.031*	
H1	0.8424	0.2944	0.1094	0.031*	
N13	0.9956 (3)	0.5987 (7)	0.1274 (2)	0.0203 (9)	
N22	0.8540 (3)	1.1387 (10)	0.2764 (3)	0.0407 (14)	
H3	0.8445	1.0060	0.2554	0.049*	
H4	0.8165	1.2039	0.2934	0.049*	
N23	0.9844 (3)	1.1572 (8)	0.2589 (2)	0.0233 (10)	
C12	0.9448 (3)	0.4289 (9)	0.1092 (2)	0.0211 (11)	
C14	1.0663 (3)	0.5662 (9)	0.1040 (2)	0.0211 (11)	
C15	1.0682 (3)	0.3592 (9)	0.0687 (2)	0.0211 (11)	
C16	1.1336 (4)	0.3037 (10)	0.0417 (3)	0.0260 (12)	
H16	1.1348	0.1632	0.0182	0.031*	
C17	1.1968 (4)	0.4568 (10)	0.0498 (3)	0.0291 (12)	
H17	1.2419	0.4215	0.0316	0.035*	
C18	1.1949 (4)	0.6625 (10)	0.0843 (3)	0.0289 (12)	
H18	1.2386	0.7664	0.0891	0.035*	
C19	1.1302 (3)	0.7179 (9)	0.1119 (3)	0.0234 (11)	
H19	1.1297	0.8578	0.1358	0.028*	
C22	0.9244 (3)	1.2400 (10)	0.2824 (3)	0.0265 (12)	
C24	1.0508 (3)	1.3024 (9)	0.2740 (3)	0.0225 (11)	
C25	1.0396 (4)	1.5002 (10)	0.3098 (3)	0.0271 (12)	
C26	1.1004 (4)	1.6632 (11)	0.3289 (3)	0.0367 (15)	
H26	1.0922	1.7971	0.3532	0.044*	
C27	1.1727 (4)	1.6241 (11)	0.3114 (3)	0.0412 (16)	
H27	1.2151	1.7321	0.3239	0.049*	
C28	1.1841 (4)	1.4289 (11)	0.2758 (3)	0.0349 (14)	
H28	1.2344	1.4051	0.2643	0.042*	
C29	1.1238 (3)	1.2679 (10)	0.2568 (3)	0.0257 (12)	
H29	1.1324	1.1352	0.2322	0.031*	
C1B	1.3701 (7)	0.437 (2)	−0.0513 (6)	0.025 (3)*	0.437 (11)
H1B1	1.3823	0.2739	−0.0424	0.037*	0.437 (11)
H1B2	1.3935	0.4888	−0.0884	0.037*	0.437 (11)
H1B3	1.3118	0.4586	−0.0634	0.037*	0.437 (11)
C2B	1.4053 (8)	0.5766 (19)	0.0114 (6)	0.048 (4)*	0.437 (11)
C3B	1.3956 (8)	0.8295 (19)	0.0124 (7)	0.031 (3)*	0.437 (11)
H3B1	1.4224	0.8892	0.0566	0.046*	0.437 (11)
H3B2	1.3384	0.8679	0.0034	0.046*	0.437 (11)
H3B3	1.4196	0.8989	−0.0222	0.046*	0.437 (11)
O4B	1.4376 (8)	0.471 (2)	0.0598 (6)	0.065 (4)*	0.437 (11)
C1A	1.3839 (5)	0.3211 (14)	−0.0243 (4)	0.015 (2)*	0.563 (11)
H1A1	1.3826	0.2663	−0.0699	0.022*	0.563 (11)
H1A2	1.3316	0.2945	−0.0135	0.022*	0.563 (11)
H1A3	1.4254	0.2376	0.0078	0.022*	0.563 (11)
C2A	1.4039 (13)	0.591 (3)	−0.0197 (10)	0.158 (13)*	0.563 (11)
C3A	1.3977 (5)	0.7536 (14)	0.0394 (4)	0.0124 (19)*	0.563 (11)
H3A1	1.4029	0.9136	0.0260	0.019*	0.563 (11)
H3A2	1.4408	0.7175	0.0785	0.019*	0.563 (11)
H3A3	1.3456	0.7319	0.0511	0.019*	0.563 (11)
O4A	1.437 (2)	0.641 (5)	−0.0618 (14)	0.287 (19)*	0.563 (11)

### Atomic displacement parameters (Å^2^)

**Table:**

	*U*^11^	*U*^22^	*U*^33^	*U*^12^	*U*^13^	*U*^23^
Ag1	0.0244 (2)	0.0184 (2)	0.0223 (2)	−0.00038 (16)	0.00910 (15)	−0.00495 (15)
S11	0.0323 (7)	0.0151 (6)	0.0244 (7)	−0.0027 (5)	0.0121 (6)	−0.0023 (5)
S21	0.0368 (8)	0.0349 (8)	0.0288 (8)	0.0101 (7)	0.0077 (6)	−0.0126 (6)
O1	0.034 (2)	0.031 (2)	0.034 (2)	0.0076 (19)	0.0140 (18)	0.0020 (18)
O2	0.061 (3)	0.028 (2)	0.044 (3)	−0.010 (2)	0.034 (2)	−0.006 (2)
O3	0.034 (3)	0.044 (3)	0.058 (3)	0.012 (2)	0.009 (2)	0.000 (2)
N1	0.024 (2)	0.023 (2)	0.035 (3)	−0.0036 (19)	0.008 (2)	−0.004 (2)
N12	0.029 (2)	0.018 (2)	0.032 (2)	−0.0029 (19)	0.013 (2)	−0.0028 (19)
N13	0.027 (2)	0.015 (2)	0.020 (2)	−0.0036 (18)	0.0089 (18)	−0.0026 (17)
N22	0.026 (3)	0.055 (4)	0.045 (3)	−0.005 (3)	0.017 (2)	−0.023 (3)
N23	0.022 (2)	0.026 (2)	0.022 (2)	0.0037 (19)	0.0043 (18)	−0.0069 (19)
C12	0.033 (3)	0.014 (2)	0.018 (2)	0.001 (2)	0.010 (2)	0.0057 (19)
C14	0.030 (3)	0.017 (2)	0.018 (2)	0.002 (2)	0.007 (2)	0.0011 (19)
C15	0.031 (3)	0.015 (2)	0.019 (2)	−0.004 (2)	0.008 (2)	0.0008 (19)
C16	0.038 (3)	0.019 (3)	0.023 (3)	0.002 (2)	0.013 (2)	0.000 (2)
C17	0.032 (3)	0.029 (3)	0.030 (3)	0.003 (2)	0.016 (2)	0.002 (2)
C18	0.032 (3)	0.029 (3)	0.029 (3)	−0.008 (2)	0.012 (2)	−0.002 (2)
C19	0.029 (3)	0.020 (3)	0.021 (3)	−0.003 (2)	0.006 (2)	−0.005 (2)
C22	0.024 (3)	0.031 (3)	0.025 (3)	0.004 (2)	0.006 (2)	−0.009 (2)
C24	0.026 (3)	0.021 (3)	0.018 (2)	0.004 (2)	0.000 (2)	−0.001 (2)
C25	0.031 (3)	0.024 (3)	0.023 (3)	0.006 (2)	−0.002 (2)	−0.003 (2)
C26	0.049 (4)	0.024 (3)	0.031 (3)	0.002 (3)	−0.004 (3)	−0.003 (2)
C27	0.039 (4)	0.031 (3)	0.046 (4)	−0.011 (3)	−0.007 (3)	0.003 (3)
C28	0.026 (3)	0.031 (3)	0.046 (4)	−0.002 (3)	0.004 (3)	0.006 (3)
C29	0.026 (3)	0.023 (3)	0.026 (3)	0.002 (2)	0.002 (2)	0.001 (2)

### Geometric parameters (Å, °)

**Table:**

Ag1—N13	2.130 (4)	C19—H19	0.9500
Ag1—N23	2.127 (4)	C24—C29	1.384 (8)
Ag1—S11^i^	3.2261 (15)	C24—C25	1.400 (7)
S11—C12	1.758 (5)	C25—C26	1.397 (9)
S11—C15	1.736 (5)	C26—C27	1.379 (10)
S21—C22	1.759 (6)	C26—H26	0.9500
S21—C25	1.735 (6)	C27—C28	1.385 (9)
O1—N1	1.264 (6)	C27—H27	0.9500
O2—N1	1.240 (6)	C28—C29	1.383 (8)
O3—N1	1.233 (6)	C28—H28	0.9500
N12—C12	1.321 (7)	C29—H29	0.9500
N12—H2	0.8800	C1B—C2B	1.522 (9)
N12—H1	0.8800	C1B—H1B1	0.9800
N13—C12	1.313 (7)	C1B—H1B2	0.9800
N13—C14	1.406 (7)	C1B—H1B3	0.9800
N22—C22	1.322 (8)	C2B—O4B	1.189 (7)
N22—H3	0.8800	C2B—C3B	1.481 (9)
N22—H4	0.8800	C3B—H3B1	0.9800
N23—C22	1.318 (7)	C3B—H3B2	0.9800
N23—C24	1.394 (7)	C3B—H3B3	0.9800
C14—C19	1.387 (8)	C1A—C2A	1.607 (18)
C14—C15	1.408 (7)	C1A—H1A1	0.9800
C15—C16	1.390 (8)	C1A—H1A2	0.9800
C16—C17	1.381 (8)	C1A—H1A3	0.9800
C16—H16	0.9500	C2A—O4A	1.160 (19)
C17—C18	1.393 (8)	C2A—C3A	1.553 (18)
C17—H17	0.9500	C3A—H3A1	0.9800
C18—C19	1.385 (8)	C3A—H3A2	0.9800
C18—H18	0.9500	C3A—H3A3	0.9800
			
N13—Ag1—N23	171.84 (17)	N23—C24—C25	114.4 (5)
N23—Ag1—S11^i^	92.96 (13)	C26—C25—C24	121.6 (6)
N13—Ag1—S11^i^	86.30 (12)	C26—C25—S21	128.0 (5)
C15—S11—C12	89.8 (3)	C24—C25—S21	110.4 (4)
C25—S21—C22	88.9 (3)	C27—C26—C25	118.0 (6)
O1—N1—O2	118.8 (5)	C27—C26—H26	121.0
O1—N1—O3	119.3 (5)	C25—C26—H26	121.0
O2—N1—O2	121.8 (5)	C26—C27—C28	120.7 (6)
C12—N12—H2	120.0	C26—C27—H27	119.7
C12—N12—H1	120.0	C28—C27—H27	119.7
H2—N12—H1	120.0	C29—C28—C27	121.3 (6)
C12—N13—C14	111.6 (4)	C29—C28—H28	119.4
C12—N13—Ag1	125.6 (4)	C27—C28—H28	119.4
C14—N13—Ag1	122.6 (3)	C28—C29—C24	119.2 (5)
C22—N22—H3	120.0	C28—C29—H29	120.4
C22—N22—H4	120.0	C24—C29—H29	120.4
H3—N22—H4	120.0	C2B—C1B—H1B1	109.5
C22—N23—C24	111.1 (5)	C2B—C1B—H1B2	109.5
C22—N23—Ag1	127.2 (4)	H1B1—C1B—H1B2	109.5
C24—N23—Ag1	120.9 (3)	C2B—C1B—H1B3	109.5
N13—C12—N12	125.1 (5)	H1B1—C1B—H1B3	109.5
N13—C12—S11	114.6 (4)	H1B2—C1B—H1B3	109.5
N12—C12—S11	120.2 (4)	O4B—C2B—C3B	122.3 (12)
C19—C14—N13	126.2 (5)	O4B—C2B—C1B	116.5 (11)
C19—C14—C15	119.7 (5)	C3B—C2B—C1B	121.1 (11)
N13—C14—C15	114.1 (5)	C2B—C3B—H3B1	109.5
C16—C15—C14	121.0 (5)	C2B—C3B—H3B2	109.5
C16—C15—S11	129.2 (4)	H3B1—C3B—H3B2	109.5
C14—C15—S11	109.9 (4)	C2B—C3B—H3B3	109.5
C17—C16—C15	118.7 (5)	H3B1—C3B—H3B3	109.5
C17—C16—H16	120.7	H3B2—C3B—H3B3	109.5
C15—C16—H16	120.7	C2A—C1A—H1A1	109.5
C16—C17—C18	120.5 (5)	C2A—C1A—H1A2	109.5
C16—C17—H17	119.8	H1A1—C1A—H1A2	109.5
C18—C17—H17	119.8	C2A—C1A—H1A3	109.5
C19—C18—C17	121.1 (5)	H1A1—C1A—H1A3	109.5
C19—C18—H18	119.4	H1A2—C1A—H1A3	109.5
C17—C18—H18	119.4	O4A—C2A—C3A	124 (2)
C18—C19—C14	119.0 (5)	O4A—C2A—C1A	109 (2)
C18—C19—H19	120.5	C3A—C2A—C1A	126.1 (16)
C14—C19—H19	120.5	C2A—C3A—H3A1	109.5
N23—C22—N22	124.8 (5)	C2A—C3A—H3A2	109.5
N23—C22—S21	115.1 (4)	H3A1—C3A—H3A2	109.5
N22—C22—S21	120.1 (4)	C2A—C3A—H3A3	109.5
C29—C24—N23	126.3 (5)	H3A1—C3A—H3A3	109.5
C29—C24—C25	119.2 (5)	H3A2—C3A—H3A3	109.5
			
S11^i^—Ag1—N13—C12	−117.6 (4)	C15—C14—C19—C18	0.2 (8)
S11^i^—Ag1—N13—C14	68.7 (4)	C24—N23—C22—N22	−179.3 (6)
S11^i^—Ag1—N23—C22	103.0 (5)	Ag1—N23—C22—N22	11.1 (9)
S11^i^—Ag1—N23—C24	−65.7 (4)	C24—N23—C22—S21	1.0 (6)
C14—N13—C12—N12	−179.2 (5)	Ag1—N23—C22—S21	−168.6 (3)
Ag1—N13—C12—N12	6.4 (7)	C25—S21—C22—N23	−1.2 (5)
C14—N13—C12—S11	2.5 (6)	C25—S21—C22—N22	179.1 (5)
C15—S11—C12—N13	−2.1 (4)	C22—N23—C24—C29	−180.0 (5)
C15—S11—C12—N12	179.6 (5)	Ag1—N23—C24—C29	−9.6 (7)
C12—N13—C14—C19	177.0 (5)	C22—N23—C24—C25	−0.2 (7)
Ag1—N13—C14—C19	−8.5 (7)	Ag1—N23—C24—C25	170.2 (4)
C12—N13—C14—C15	−1.7 (6)	C29—C24—C25—C26	−0.2 (8)
Ag1—N13—C14—C15	172.8 (3)	N23—C24—C25—C26	179.9 (5)
C19—C14—C15—C16	0.4 (8)	C29—C24—C25—S21	179.1 (4)
N13—C14—C15—C16	179.2 (5)	N23—C24—C25—S21	−0.7 (6)
C19—C14—C15—S11	−178.6 (4)	C22—S21—C25—C26	−179.7 (6)
N13—C14—C15—S11	0.2 (6)	C22—S21—C25—C24	1.0 (4)
C12—S11—C15—C16	−177.9 (5)	C24—C25—C26—C27	−0.1 (9)
C12—S11—C15—C14	1.0 (4)	S21—C25—C26—C27	−179.3 (5)
C14—C15—C16—C17	−0.6 (8)	C25—C26—C27—C28	0.2 (9)
S11—C15—C16—C17	178.2 (4)	C26—C27—C28—C29	0.0 (10)
C15—C16—C17—C18	0.1 (8)	C27—C28—C29—C24	−0.3 (9)
C16—C17—C18—C19	0.5 (9)	N23—C24—C29—C28	−179.8 (5)
C17—C18—C19—C14	−0.7 (9)	C25—C24—C29—C28	0.5 (8)
N13—C14—C19—C18	−178.4 (5)		

Symmetry codes: (i) *x*, *y*+1, *z*.

### Hydrogen-bond geometry (Å, °)

**Table:**

*D*—H···*A*	*D*—H	H···*A*	*D*···*A*	*D*—H···*A*
N12—H2···O1	0.88	1.99	2.851 (6)	165
N12—H1···O2^ii^	0.88	2.07	2.889 (6)	155
N22—H3···O1	0.88	2.12	2.959 (7)	158
N22—H4···O1^iii^	0.88	2.11	2.955 (6)	162

Symmetry codes: (ii) *x*, *y*−1, *z*; (iii) −*x*+3/2, *y*+1/2, −*z*+1/2.
